# Comparative Analysis of Root Canal Microbiota in Patients with Diabetes and Systemically Healthy Individuals: A Pilot Next-Generation Sequencing Study

**DOI:** 10.3390/jcm14186643

**Published:** 2025-09-20

**Authors:** Nazife Maide Dayıcan, Sevinç Aktemur Türker

**Affiliations:** Department of Endodontics, Faculty of Dentistry, Zonguldak Bülent Ecevit University, 67100 Zonguldak, Turkey; maidedayican@gmail.com

**Keywords:** diabetes mellitus, root canal infection, next generation sequencing, apical periodontitis, endodontic microbiology

## Abstract

**Objectives:** The aim of this study is to assess the influence of diabetes mellitus on the microbial flora involved in root canal infections through a comparative analysis with that of systemically healthy individuals. **Methods:** A total of 39 participants, including 21 patients with diabetes mellitus and 18 systemically healthy individuals (controls), were enrolled in the study. In the diabetic group, 12 teeth were diagnosed with secondary/persistent endodontic infections (SEIs) and 9 with primary endodontic infections (PEIs). In the healthy group, 12 teeth presented with SEIs and 6 with PEIs. Root canal samples were obtained using sterile paper points. The V3–V4 hypervariable regions of 16S rDNA from both sample types were amplified and sequenced using the Illumina MiSeq platform. Microbial richness and diversity were assessed using alpha diversity indices and beta diversity metrics. **Results:** Faith’s Phylogenetic Diversity showed a significant difference between diabetic patients with SEIs and healthy individuals with PEIs (*p* = 0.02). Both weighted and unweighted UniFrac beta diversity analyses indicated significant differences in microbial composition and phylogenetic structure between diabetic patients with SEIs and healthy individuals with PEIs (*p* = 0.01 and *p* = 0.02, respectively). Within the diabetic patient group, significant differences were observed between SEI and PEI groups based on alpha (Fisher’s alpha, *p* = 0.04) and beta diversity analyses (Bray–Curtis and Weighted UniFrac *p* = 0.02 and *p* = 0.01, respectively). **Conclusions:** Diabetic patients showed different microbial profiles compared to healthy individuals.

## 1. Introduction

Apical periodontitis (AP) is a chronic inflammatory disease which is triggered by the polymicrobial colonization of the root canal. It occurs when microorganisms or their toxins and virulence factors reach the periradicular area [1]. It is characterized by the resorption and degradation of the alveolar bone in the periapical area. AP can exist in an acute or chronic form, depending on the severity of the bacterial infection and systemic host factors such as the production of inflammatory mediators [2]. In addition to microbial and immunological determinants, host genetic variations, particularly in genes regulating immune and inflammatory responses, have also been suggested to influence individual susceptibility to AP [3].

The current literature indicates that there is a bidirectional association between apical periodontitis and systemic diseases [4,5]. Type I and II diabetes mellitus are among these systemic diseases; they are characterized by hyperglycemia resulting from insufficient insulin secretion, impaired insulin action, or a combination of both. Prolonged and uncontrolled hyperglycemia can result in vascular complications involving both large and small blood vessels, leading to numerous structural and metabolic alterations, including those affecting oral tissues. Inadequate glycemic control significantly raises the risk of oral health issues such as dental caries, tooth loss, periodontal disease, pulp necrosis, and apical periodontitis [6]. Current research supports the hypothesis that a common pathophysiological link may exist between apical periodontitis and diabetes mellitus [7,8]. Previous studies have established a direct link between diabetes and periapical lesions, showing that diabetic patients exhibit a higher prevalence and larger size of periapical lesions compared to non-diabetic individuals [9,10]. However, the number of studies examining the microbial flora of endodontic infections in diabetic patients remains limited [11,12,13,14,15]. Those investigations have mainly focused on the detection of specific bacterial species.

Fouad [16] suggested that diabetic patients may be more prone to persistent infections, possibly due to different microbial profiles in teeth with pulp necrosis. Specifically, diabetic patients might harbor a more virulent or pathogenic microbiota within the root canal system. This hypothesis aligns with the broader understanding that systemic conditions like diabetes can alter host immune responses and microbiome composition. However, despite this plausible link, there is currently a lack of comprehensive studies that directly compare the endodontic microbiota of diabetic patients with that of systemically healthy individuals.

To date, no study has investigated the microbial diversity between primary and secondary/persistent root canal infections in diabetic patients. Therefore, this study aimed to assess the influence of diabetes mellitus on the microbial flora involved in root canal infections through a comparative analysis with that of systemically healthy individuals by employing next-generation sequencing techniques. This study had two null hypotheses. The first was that there would be no difference in the microbial communities of root canal infections between diabetic patients and systemically healthy individuals. The second was that the microbial communities in primary and secondary/persistent root canal infections of diabetic patients do not differ.

## 2. Materials and Methods

Ethical approval for this study was obtained from the Non-Interventional Clinical Research Ethics Committee of the University (Approval No: 2023/18) and informed consent was obtained from all patients prior to participation in the study. The study protocol was registered on www.clinicaltrials.gov (ID: NCT07047027). Teeth diagnosed with chronic apical periodontitis and presenting with distinct periapical radiolucency on radiographic evaluation were selected from patients aged between 18 and 65 years. A total of 39 participants were enrolled, including 21 patients with diabetes mellitus and 18 systemically healthy controls.

The inclusion criteria for patient selection were as follows: patients in the diabetic group were required to have a diagnosis of type 2 diabetes mellitus with no other systemic diseases; patients in the control group had to be systemically healthy with no history of any systemic illness. All participants had to not have used antibiotics within the past three months, could not be pregnant, and had to have teeth with intact coronal restorations that prevent direct pulp exposure to the oral environment. Only asymptomatic teeth diagnosed with chronic apical periodontitis and a Periapical Index (PAI) score of 3 or higher were included [17]. The exclusion criteria were as follows: teeth with extensive coronal destruction that would prevent rubber dam isolation; presence of pain, swelling, or sinus tract; presence of root or crown fractures; and periodontal pockets deeper than 3 mm.

The teeth included in the study from both the control and diabetic groups were classified as having either primary endodontic infections (PEIs) or secondary/persistent endodontic infections (SEIs). Teeth with PEIs were defined as previously untreated teeth diagnosed with asymptomatic chronic apical periodontitis and a Periapical Index (PAI) score of ≥3. Teeth with SEIs were defined as previously root canal-treated teeth with a root canal filling ending 0–2 mm short of the radiographic apex, also diagnosed with asymptomatic chronic apical periodontitis, and with a PAI score of ≥3. In the diabetic group, 12 teeth were diagnosed with SEIs and 9 with PEIs. In the control group, 12 teeth were diagnosed with SEIs and 6 with PEIs.

### 2.1. Microbial Sampling from Root Canals

Microbial sampling from root canals was carried out under strict aseptic conditions. All procedures followed during this phase were based on the protocol described by Amaral et al. [18]. Initially, patients rinsed their mouths with a 0.12% chlorhexidine mouthwash for 1 min. Supragingival plaque and debris were then removed using an ultrasonic scaler. The teeth were isolated with a rubber dam, and the crown surfaces were disinfected by wiping with 3% hydrogen peroxide (H_2_O_2_) followed by 5.25% sodium hypochlorite (NaOCl). To neutralize the effect of sodium hypochlorite, 5% sodium thiosulfate (Na_2_S_2_O_3_) was applied. This disinfection protocol was performed both before and after access cavity preparation.

Access cavities were prepared under sterile saline irrigation using sterile burs. After cavity preparation, samples were collected from the inner walls of the access cavity using sterile paper points to check for sterility. These control samples were placed into Eppendorf tubes containing phosphate-buffered saline (PBS) and stored at −80 °C. Teeth were included in the study only after PCR analysis confirmed the absence of bacterial contamination in the control samples.

In teeth diagnosed with PEIs, sampling procedures were performed as follows: Working length determination was performed using a size #10 K-file inserted into the root canal to establish a glide path, in conjunction with an electronic apex locator (Ai-Pex, Woodpecker Medical Instrument Co., Ltd., Guilin, China). The “00” reading on the apex locator was considered the working length, and a confirmatory periapical radiograph was taken to verify accuracy. Subsequently, 1 mL of sterile saline solution was introduced into the canal, and three sterile #15 paper points (DiaDent, Group International, Cheongju, Republic of Korea) were sequentially inserted to working length for 30 s each to absorb root canal contents. In multi-rooted teeth, microbial samples were taken from the root associated with the largest periapical lesion. In teeth diagnosed with secondary/persistent apical periodontitis, after removal of previous root canal filling materials using ProTaper Universal Retreatment files (Dentsply Sirona, Ballaigues, Switzerland) at 500 rpm and 2 N·cm torque, the same protocol as mentioned for teeth with PEIs was carried out. Residual filling materials and paper points were inserted into Eppendorf tubes containing PBS and stored at –80 °C until further analysis.

### 2.2. DNA Extraction and Sequencing

Microbial DNA was extracted using the QIAamp DNA Stool Mini Kit (QIAGEN, Hilden, Germany) according to the manufacturer’s instructions. To assess DNA quality, the purity of the isolated DNA was measured using a NanoDrop spectrophotometer (Thermo Fisher Scientific, Waltham, MA, USA) by evaluating the A260/A280 and A260/A230 ratios. DNA concentration was determined using a Qubit 4.0 fluorometer (Thermo Fisher Scientific, Waltham, MA, USA) with the Qubit dsDNA HS Assay Kit. DNA integrity was verified via 1% agarose gel electrophoresis.

For 16S rRNA gene amplification, the V3–V4 region of the bacterial genome was targeted using primers 341F (5′-CCTACGGGNGGCWGCAG-3′) and 805R (5′-GACTACHVGGGTATCTAATCC-3′). PCR amplification was performed using the 2X KAPA HiFi HotStart ReadyMix. The thermal cycling protocol included an initial denaturation at 95 °C for 3 min, followed by 25 cycles of denaturation at 95 °C for 30 s, annealing at 55 °C for 30 s, extension at 72 °C for 30 s, and a final extension at 72 °C for 5 min.

PCR products were purified using AMPure XP magnetic beads (Beckman Coulter, Brea, CA, USA). Dual-index adapters were added to the purified amplicons using the Nextera XT Index Kit v2 (Illumina, San Diego, CA, USA). The indexed PCR products were again purified with AMPure XP beads, and their concentrations were quantified using Qubit. Libraries were pooled in equimolar concentrations and sequenced on the Illumina MiSeq platform using a 2 × 250 bp paired-end configuration.

### 2.3. Bioinformatic Analyses

Raw sequence data were analyzed using the QIIME 2 platform. In the initial stage, demultiplexing was performed to separate reads for each sample. Quality filtering, error correction, and chimera removal were carried out using the DADA2 algorithm. Low-quality bases (Q-score < 25) and short reads (<200 bp) were removed during this process.

Amplicon sequence variants (ASVs) were identified using the DADA2 pipeline. Taxonomic classification of each ASV was performed using a naïve Bayes classifier trained on the SILVA 138.1 16S rRNA gene reference database. Taxonomic assignments were obtained at the phylum and genus levels.

Alpha diversity was assessed using species richness index Chao1, and diversity indices including Shannon, Simpson, Fisher’s alpha, and Faith’s Phylogenetic Diversity (Faith’s PD). Intergroup comparisons of alpha diversity were conducted using the Kruskal–Wallis test. For beta diversity, distance metrics such as Bray–Curtis, Jaccard, Weighted UniFrac, and Unweighted UniFrac were used. Principal Coordinate Analysis (PCoA) was performed. Differences in beta diversity between groups were assessed using PERMANOVA tests. Differentially abundant taxa contributing to compositional differences among groups were identified through LEfSe (Linear Discriminant Analysis Effect Size) analysis.

## 3. Results

The microbial DNA analysis of the 39 samples revealed that all samples were positive for 16S rRNA. Table 1 presents data on the 21 diabetic patients, including tooth number, type of infection, gender, age, HbA1c levels, and duration of diabetes. Table 2 presents data on the 18 systemically healthy individuals, including tooth number, gender, age, and type of infection.

Demographic data were compared using the Kruskal–Wallis test for continuous variables and the chi-square test for categorical variables. A statistically significant difference in age distribution was observed between the diabetic PEI and healthy PEI groups (*p* = 0.018). However, no significant differences were found between the groups regarding gender distribution (*p* = 0.195) or the type of treated teeth (*p* = 0.497).

Within the diabetic group, no statistically significant differences were found between PEI and SEI subgroups in terms of age (*p* = 0.193), HbA1c levels (*p* = 0.837), or duration of diabetes diagnosis (*p* = 0.754). Likewise, no significant differences were observed regarding the type of treated teeth (*p* = 0.156) or gender distribution (*p* = 0.080).

When comparing the diabetic SEI and healthy SEI groups, a statistically significant difference was found in age distribution (*p* = 0.002), whereas no significant differences were observed in terms of treated tooth type (*p* = 0.217) or gender distribution (*p* = 0.131).

In the healthy group, comparisons between PEI and SEI subgroups showed no statistically significant differences in age (*p* = 0.102), type of treated teeth (*p* = 0.230), or gender distribution (*p* = 0.316).

### 3.1. Microbial Analysis

**Figure 1:**
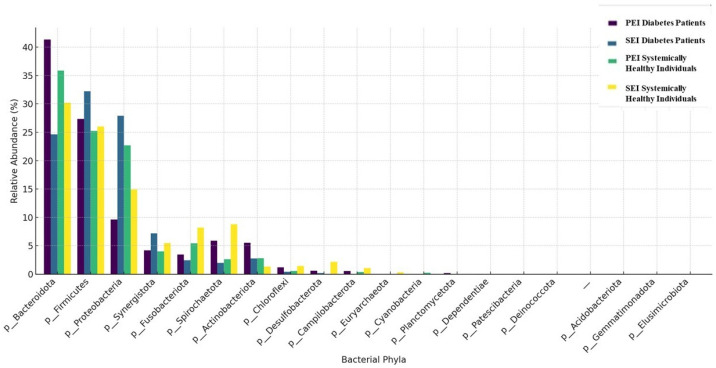
Relative abundance of microbial communities at the phylum level across groups. Only phyla with a relative abundance greater than 1% are shown.

**Figure 2:**
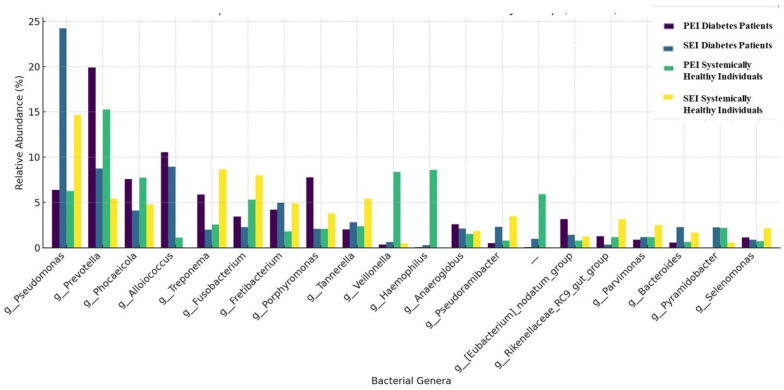
Relative abundance of microbial communities at the genus level across groups. Only phyla with a relative abundance greater than 1% are shown.

The taxonomic composition of the samples at the phylum and genus levels is shown in Figure 1 and Figure 2, respectively. A total of 22 phyla and 237 genera were detected in the diabetes patients. In diabetic patients with both PEIs and SEIs, the most commonly identified phyla were *Bacteroidetes*, *Firmicutes*, and *Proteobacteria*. The most frequently identified genera in PEI samples were *Prevotella*, *Alloiococcus*, *Porphyromonas*, and *Phocaeicola*. In SEI samples, the dominant genera were *Pseudomonas*, *Alloiococcus*, *Prevotella*, and *Fretibacterium*.

A total of 18 phyla and 158 genera were identified in systemically healthy individuals. In systemically healthy individuals with PEIs and SEIs, the most abundant phyla were *Bacteroidetes*, *Firmicutes*, and *Proteobacteria*. The most frequently identified genera in PEI samples from systemically healthy individuals were *Prevotella*, *Veillonella*, *Haemophilus*, *Phocaeicola*, and *Pseudomonas*. The most prevalent genera in SAP samples were *Pseudomonas*, *Treponema*, *Fusobacterium*, and *Prevotella*.

Archaea were detected in five samples. Three of these samples were from diabetic patients diagnosed with SEIs. In these three samples, *Methanobrevibacter* was identified at the genus level, with a relative abundance of 0.20%. Among the two samples obtained from systemically healthy individuals, one was from a patient with SEI, and *Methanobrevibacter* was identified at the genus level, with a relative abundance of 1.97%. The other sample, from a patient with PEI, showed the presence of *Methanobrevibacter wolinii* at the species level, with a relative abundance of 0.26%.

*Enterococcus* was detected in four samples out of the total, which were obtained from diabetic patients. Two of these samples belonged to patients with SAP, with relative abundances of 12.25% and 0.29%. The remaining two samples were from diabetic patients with PEI, showing relative abundances of 1.04% and 0.13%.

### 3.2. Comparison of Alpha Diversity Indices Among Groups

Statistically significant alterations in microbial communities were identified between diabetic patients and systemically healthy individuals. Faith’s Phylogenetic Diversity showed a significant difference between diabetic patients with SEIs and healthy individuals with PEIs (*p* = 0.02). Fisher alpha demonstrated a statistically significant difference between PEIs and SEIs in diabetic patients (*p* = 0.04).

**Figure 3:**
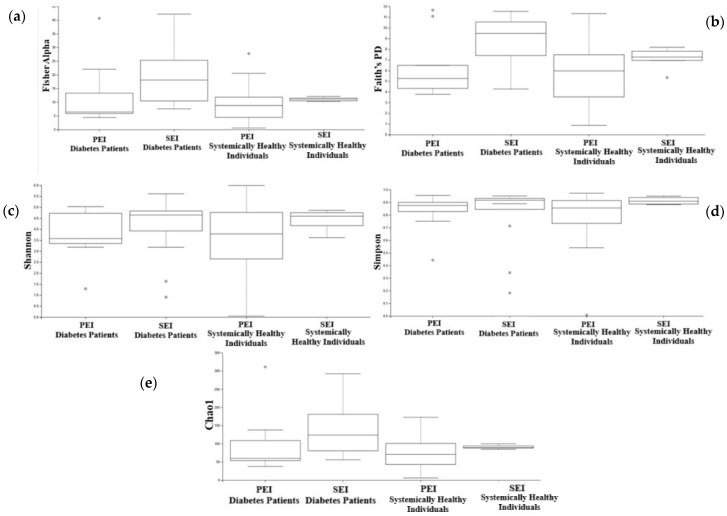
Microbial diversity based on (**a**) Fisher’s alpha index, with a significant difference observed between the groups (*p* = 0.04); (**b**) Faith’s PD index, with significant differences found between primary and persistent apical periodontitis in diabetic patients (*p* = 0.04); (**c**) the Shannon index, with no significant differences observed in microbial community diversity between the groups (*p* = 0.63); (**d**) the Simpson index, with no significant differences observed in microbial community diversity between the groups (*p* = 0.35); and (**e**) microbial richness based on the Chao1 index, with no significant differences in microbial richness found between the groups (*p* = 0.10). The black dots denote outliers, defined as values outside the whisker range.

The Shannon and Simpson indices showed no statistically significant differences in microbial diversity between the groups (*p* = 0.63 and *p*= 0.35, respectively). Similarly, the Chao1 index indicated no significant differences in microbial richness (*p* = 0.10) (Figure 3).

### 3.3. Comparison of Beta Diversity Indices Among Groups

**Figure 4:**
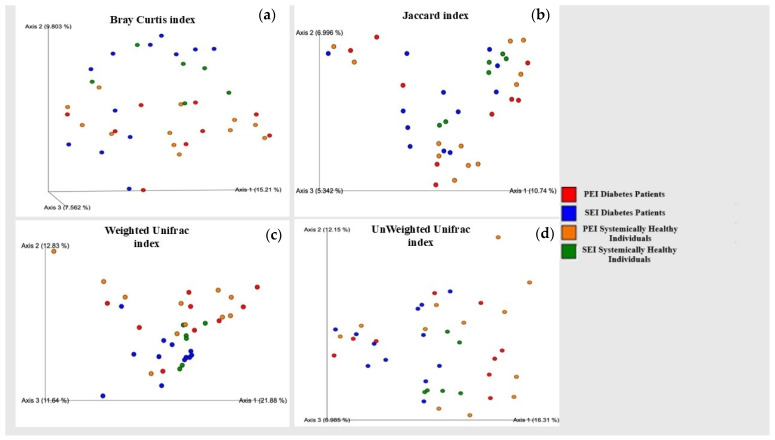
Principal coordinate analysis of the microbiota community structures of groups based on the (**a**) Bray–Curtis, (**b**) Jaccard, (**c**) weighted UniFrac, and (**d**) unweighted UniFrac distances.

The Weighted UniFrac and the Unweighted UniFrac distance matrixes revealed significant phylogenetic differences between diabetic patients with SEIs and systemically healthy individuals with PEIs (*p* = 0.01, *p* = 0.02, respectively). Similarly, the Weighted UniFrac distance matrix and the Bray–Curtis index revealed significant differences between the microbial communities of diabetic patients with PEIs and SEIs (*p* = 0.02, *p* = 0.02), while no significant differences were observed between the groups using the Jaccard index (*p* = 0.23). These findings were further confirmed by PCoAs, which demonstrated clear separations between the groups (Figure 4).

For systemically healthy individuals, no statistically significant differences were observed in any of the alpha or beta diversity indices between PEIs and SEIs (*p* > 0.05).

**Figure 5:**
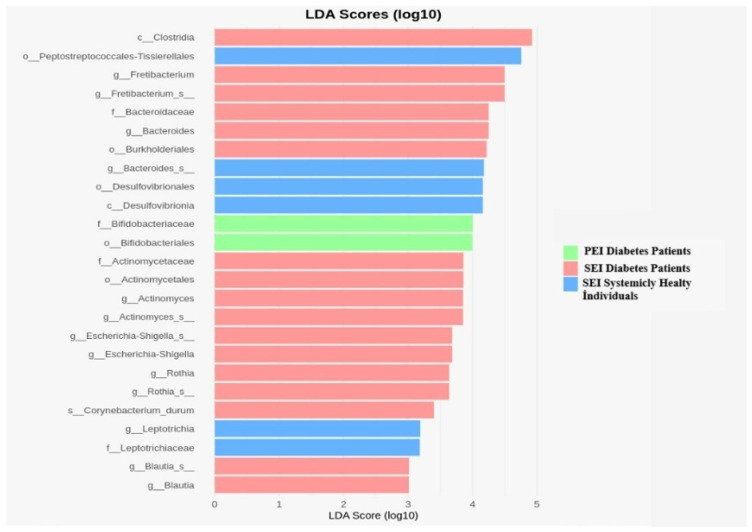
The horizontal bar graphs indicate differentially abundant bacterial taxa in groups.

According to LEfse analysis, in diabetic patients with SEIs, the following biomarkers were significantly more abundant: p. *Synergistota*, g. *Fretibacterium*, g. *Bacteroides*, g. *Actinomyces*, g. *Escherichia-Shigella*, g. *Rothia*, and g. *Blautia* (LDA > 1). In diabetic patients with PEIs, biomarkers including f. *Bifidobacteraceae* and o. *Bifidobacteriales* were identified as significant differentiators. In systemically healthy individuals with SEIs, the biomarkers p. *Spirochaetota*, g. *Bacteroides*, and g. *Leptotrichiaceae* were found to be significantly more abundant (LDA > 1), as shown in Figure 5.

## 4. Discussion

While the role of microbial etiology in apical periodontitis is well established, the influence of systemic conditions such as diabetes on the microbial profiles of endodontic infections remains inadequately explored. To our knowledge, this is the first study to specifically identify and compare the microbiota associated with different forms of root canal infections in a diabetic population. The results of the present study reveal important insights into the microbial dynamics of root canal infections in diabetic patients. The present study revealed 18 phyla and 158 genera for PEIs and SEIs in systemically healthy individuals, whereas 22 phyla and 237 genera were identified in diabetic patients. In both groups, the most abundant phyla were *Firmicutes*, *Bacteroidetes*, *and Proteobacteria.* This result is in line with several previous studies [19,20].

The most frequently detected genera in the PEI group were *Prevotella*, *Alloiococcus*, *Porphyromonas*, and *Phocaeicola*, whereas the dominant genera in the SEI group were *Pseudomonas*, *Alloiococcus*, *Prevotella*, and *Fretibacterium*. *Alloiococcus* was identified as the second most abundant genus in both PEI and SEI samples from diabetic patients. *Alloiococcus* is a genus of Gram-positive and nonmotile bacteria from the phylum of *Bacillota*. Notably, this bacterial genus has not been reported in previous studies investigating the microbiota of root canal infections. However, *Alloiococcus* was detected in a study by Tang et al. [21] which analyzed the oral microbiota of diabetic patients using saliva samples. Despite this observation, the association between *Alloiococcus* and both diabetes mellitus and root canal infections remains unclear. Therefore, further studies are needed to elucidate the potential role of this genus in the pathogenesis of endodontic infections in diabetic patients.

Although bacteria are the primary contributors to root canal infections, recent discussions have raised the possibility that archaea, a separate domain of microorganisms, may also play a role in the development of endodontic infections. In the present study, *Methanobrevibacter* was detected in 6 out of 39 samples. Among these, four samples were obtained from diabetes patients with SEIs, while two samples were from systemically healthy individuals with PEIs. One of these two samples was identified at the species level as *Methanobrevibacter wolinii.* To date, the presence of *Methanobrevibacter wolinii* in endodontic infections has only been documented in a single previous study [22], highlighting its rare detection and the need for further investigation into its potential role in endodontic pathology.

In the present study, *Enterococcus* was detected in four samples out of all samples, which were obtained from 21 diabetic patients. Two of these samples belonged to patients with SEIs (17%), with relative abundances of 12.25% and 0.29%. The remaining two samples were from diabetic patients with PEIs (22%), showing relative abundances of 1.04% and 0.13%. In a study by Fouad et al. [12], the presence of *Enterococcus* spp. in persistent endodontic infections was investigated using PCR analysis. Among 37 patients, *Enterococcus* spp. was detected in 8 cases (22%). Notably, 6 of these 37 patients were diagnosed with diabetes mellitus, and *Enterococcus* spp. was identified in 2 of the 6 diabetic patients (33%). *Enterococcus* does not seem to be a predominant genus in most studies, given its relatively low abundance.

According to the results of the present study, while alpha diversity indices such as Shannon, Simpson, Fisher’s, and Chao1 indicated no statistically significant differences in overall microbial richness and diversity between diabetic patients and healthy individuals, a significant difference was detected in Faith’s PD index, suggesting that although the overall number and distribution of microbial taxa were similar between the groups, the phylogenetic breadth of the microbial communities differed. This finding may indicate a shift in the evolutionary relationships of the microbial taxa present in diabetic individuals, potentially reflecting alterations in the composition of functionally distinct lineages. The discrepancy between taxonomic richness (as measured by Chao1) and phylogenetic richness (as measured by Faith’s PD) underscores the importance of incorporating multiple diversity metrics when assessing the microbial ecology of systemic conditions such as diabetes.

Based on the results, while alpha diversity indices such as Shannon, Simpson, Faith’s PD, and Chao1 indicated no statistically significant differences in overall microbial richness and diversity between PEIs and SEIs in diabetic patients, a significant difference was detected in Fisher’s alpha index. This suggests that the phylogenetic structure of microbial communities may differ between primary and persistent apical periodontitis in diabetic patients, even when species richness appears similar. These findings imply that diabetes may influence the ecological complexity of endodontic infections over time or following treatment failure. Beta diversity metrics further supported this observation.

Both Weighted and Unweighted UniFrac distance metrics demonstrated significant phylogenetic differences between diabetic patients with SEIs and systemically healthy individuals with PEIs. These findings suggest that not only the presence or absence of microbial taxa (as captured by Unweighted UniFrac), but also their relative abundances (as reflected in Weighted UniFrac), vary significantly between these clinical groups. This implies that diabetes may influence the composition of endodontic microbiota in a manner that affects both the structure and abundance distribution of phylogenetically distinct microbial communities. Such shifts could be related to the systemic immune and metabolic alterations associated with diabetes, which may create a microenvironment favoring specific bacterial lineages linked to secondary endodontic infections.

While the Jaccard index, which is based on the presence or absence of taxa, showed no significant difference between groups, both Bray–Curtis and Weighted UniFrac indices, sensitive to taxonomic abundance and phylogenetic relationships, revealed significant distinctions in microbial community structure between primary and persistent infections in diabetic patients. These findings imply that while the types of microorganisms present may remain relatively stable, their relative abundances and phylogenetic compositions shift in persistent infections, potentially influenced by the diabetic microenvironment, host immune modulation, or treatment resistance.

LEfSe analysis revealed distinct microbial biomarkers associated with diabetic patients and systemically healthy individuals. In diabetic patients with SEIs, the discriminating taxa included p. *Synergistota*, g. *Fretibacterium*, g. *Bacteroides*, g. *Actinomyces*, g. *Escherichia*-*Shigella*, g. *Rothia*, and g. *Blautia*, all of which showed increased relative abundance (LDA > 1). These taxa are often associated with anaerobic infections and may reflect diabetes-related alterations in host immunity or tissue microenvironment that favor their persistence. In diabetic patients with PEIs, f. *Bifidobacteriaceae* and o. *Bifidobacteriales* were identified as key biomarkers, suggesting a possible role for facultative anaerobes or gut-associated taxa in endodontic pathology within this systemic context.

Conversely, in systemically healthy individuals with SEIs, p. *Spirochaetota*, g. *Bacteroides*, and g. *Leptotrichiaceae* emerged as prominent biomarkers (LDA > 1), indicating a different microbial signature in the absence of systemic disease. The identification of *Spirochaetota* and *Leptotrichiaceae* in this group aligns with previous reports linking these taxa to chronic oral infections and suggests that the microbial ecosystem in persistent lesions may vary substantially depending on the host’s systemic health. These findings underscore the influence of diabetes on shaping not only microbial diversity but also the dominance of specific taxa, which could serve as potential biomarkers for risk stratification and individualized therapeutic strategies in endodontic infections.

Taken together, these findings provide valuable insight into the microbiological complexity of persistent apical periodontitis in diabetic patients, emphasizing the influence of systemic metabolic dysregulation on the composition and behavior of the endodontic microbiota. The identification of distinct microbial biomarkers through LEfSe analysis in both diabetic and non-diabetic individuals suggests that systemic health status can modulate the microbial ecology within infected root canals. In particular, the presence of genera such as *Fretibacterium*, *Escherichia-Shigella*, and *Rothia* in diabetic patients with persistent lesions may point to a shift toward more pathogenic or treatment-resistant microbial communities in this population. The altered microbial profiles observed in diabetic patients may contribute to reduced treatment efficacy, delayed healing, or higher reinfection rates. As such, personalized treatment protocols—potentially including more targeted antimicrobial strategies, closer post-treatment monitoring, or adjunctive systemic management—may be warranted in this high-risk group. The lack of significant differences in microbial diversity between diabetic and systemically healthy individuals within both secondary/persistent and primary endodontic infections (SEIs and PEIs) suggests that the microbial composition in these infections may be influenced more by the nature of the infection itself rather than the systemic health status of the host. Although this study focused on taxonomic composition, the identified taxa are known to possess functional traits such as biofilm resilience, virulence factors, and persistence in root canal environments. These functional characteristics may contribute to the pathogenesis and chronicity of apical periodontitis, linking microbial composition to clinical outcomes.

Nevertheless, the present study has certain limitations that should be acknowledged. First, the sample size, particularly within subgroup analyses, may limit the generalizability of the findings. The relatively small and unequal group sizes represent a limitation of the study, although the results still provide important preliminary insights that may guide future research with larger and more balanced cohorts. Second, although 16S rRNA gene sequencing provides valuable taxonomic information, it does not offer insights into microbial functionality or metabolic activity, which could be critical in understanding pathogenicity. Future studies with larger cohorts, longitudinal designs, and integration of metatranscriptomic or metabolomics approaches would be valuable to further elucidate the role of diabetes in modulating the endodontic microbiome. Moreover, understanding the functional activity of key microbial taxa could inform enhanced disinfection protocols, the use of interim calcium hydroxide medication, and tailored follow-up schedules, translating microbiome insights into practical strategies for improving endodontic treatment outcomes in diabetic patients.

## 5. Conclusions

Within the limitations of these study findings indicate that diabetes mellitus may significantly modulate the complexity and microbial composition of the root canal system, particularly in cases of secondary endodontic infections, highlighting the necessity for individualized endodontic treatment strategies in diabetic patients

## Figures and Tables

**Table 1:** Demographic and clinical characteristics of the diabetic patients.

Sample Number	Tooth Number	Type of Infection	Gender	Age	HbA1c	Duration (Year)
1	42	PEI	Female	56	6.1	10
2	45	SEI	Male	38	6.9	20
3	12	SEI	Male	52	7	15
4	17	SEI	Male	39	7	5
7	14	PEI	Female	47	8.6	10
9	45	PEI	Female	61	6.2	3
16	11	PEI	Male	47	N/A	2
19	12	SEI	Male	41	11.2	5
20	46	SEI	Male	58	6.6	15
23	16	SEI	Male	64	N/A	4
24	17	PEI	Male	57	11.2	45
25	37	SEI	Female	50	6.5	10
26	35	PEI	Female	64	10.1	20
27	32	PEI	Female	64	N/A	10
28	25	SEI	Female	48	9.0	10
30	34	SEI	Female	35	6.5	2
34	16	SEI	Male	48	N/A	9
36	43	PEI	Female	55	6.7	2
37	11	PEI	Female	52	6.7	2
38	11	SEI	Male	57	N/A	3
39	36	SEI	Female	62	7.9	10

**Table 2:** Demographic and clinical characteristics of the systemically healthy individuals.

Sample Number	Tooth Number	Type of Infection	Age	Gender
5	21	SEI	29	Female
6	44	PEI	51	Female
8	46	PEI	38	Male
10	21	PEI	26	Female
11	47	PEI	37	Male
12	16	PEI	51	Male
13	25	SEI	29	Male
14	35	SEI	29	Female
15	14	PEI	36	Female
17	36	SEI	20	Female
18	47	PEI	65	Male
21	35	SEI	48	Female
22	42	PEI	64	Female
29	34	SEI	18	Female
31	11	PEI	31	Male
32	22	PEI	19	Female
33	35	PEI	28	Female
35	32	PEI	39	Male

## Data Availability

The original contributions presented in this study are included in the article. Further inquiries can be directed to the corresponding author (s).
